# The Influence of Stud Characteristics of Football Boots Regarding Player Injuries

**DOI:** 10.3390/ijerph20010720

**Published:** 2022-12-30

**Authors:** Alejandro Castillo-Domínguez, Marcelino Torrontegui-Duarte, Joaquín Páez-Moguer, Álvaro Gómez-del-Pino, Pablo Cervera-Garvi, Elena Mainer-Pardos, Demetrio Lozano, Jerónimo García-Romero

**Affiliations:** 1 Department of Nursing and Podiatry, Faculty of Health Sciences, University of Malaga, 29071 Malaga, Spain; 2 Health Sciences Faculty, Universidad San Jorge, Autov. A23 km 299, Villanueva de Gállego, 50830 Zaragoza, Spain; 3 Department of Human Physiology, Histology, Pathological Anatomy and Sports Physical Education, University of Malaga, 29071 Malaga, Spain

**Keywords:** football, soccer footwear, team sports, lower limb injury, stud, wellness health

## Abstract

Background: the main aim of this study was to analyze the relationship between sole pattern parameters of football boots with the frequency of injuries that occur in semiprofessional and amateur footballers. Methods: The study sample was composed of 77 male football players. All were at least 18 years old, played at least 10 h per week, gave signed informed consent to take part and properly completed the Visual Analogue Scale. This study analysed data from each player’s medical history, including age, injuries, years of practice, field type and surface condition information. Results: The visual analogic score in semiprofessional players was higher (2.05 ± 2.43) than in amateur players (1.00 ± 1.1). A total of 141 lesions were collected, equivalent to 1.81 injuries for each football player studied (*n* = 77). The result of the ROC curve indicated that the player’s years of practice could predict significantly (*p* < 0.05) the presence of lower limb injuries, with an area under the curve of 0.714. Conclusions: This study described the predictive capacity of sole pattern characteristics concerning lower limb injuries in amateur and semiprofessional footballers. Football boot variables associated with the number of studs were associated with foot and ankle overload injuries.

## 1. Introduction

Football is currently the most popular sport in the world. It has more than 270 million players licensed by the “Federation International de Football Association” (FIFA), in addition to a similar number of players who practice it without being registered with any football federation. Increased sports practice in recent years has raised the incidence of injuries during the game and training [1]. Studs are the metal or plastic mounds that cover the bottom of a football boot. This element of the shoe sole can help with traction on pitch surfaces, enhance stability and prevent footballers from sliding. It is essential to wear the right kind of stud for each surface. This not only helps gameplay but also helps prevent injuries [2].

Injuries in football include sliding tackles, runs, shots on goal, turning movements, jumps or subsequent falls [3]. Lower extremities are the most frequent location, with approximately 70% of all of the injuries caused by the practice of football [4]. In order to create an agreement that would include essential aspects of injuries in the field of football, the Injury Consensus Group grouped them into different areas, in addition to establishing the Injury Rates as a measurement system, which relates to the number of injuries compared to 1000 h of activity [3]. This stands at 30.5 for every 1000 h during official matches [5], rising to 60 per 1000 h in international competitions [1]. This injury rate is reduced to 5.8 per 1000 h during training sessions. Half of all these injuries are classified as severe, keeping footballers off the pitch for more than four weeks [5].

Injuries caused without contact prevail over those caused by contact (59% versus 41%) [3] and are mainly related to the appearance of muscle fatigue and sports footwear [2]. Thigh strain is the most common injury in professional football [6], while among the most serious are those that affect ligaments, the most affected anatomical areas being the knee and ankle, in that order. Suppose we combine the most affected area (knee) with the most frequent type of injury (ligamentous). In that case, we obtain a ligamentous injury of the knee joint, which accounts for 21.7% of all injuries caused by the practice of football. The knee’s anterior cruciate and external collateral ligaments are most commonly affected [1]. Boots play an important role in load transmission and it is logical to assume that their construction and structure can be vital in protecting the foot from possible injuries [7,8]. However, other important aspects are the sensitivity of the ball, quality of the touch, durability, weight and protection [5].

We find three main types of studs: elongated or knife studs, rounded and triangular, associated with boots for different surfaces such as artificial grass (AG) and earth and natural grass (NG), resulting in a wide range of models with different features [9]. These characteristics have a different influence on a feature catalogued as an essential property, i.e., the traction forces of the footwear [10]. AG and NG surfaces, concerning the different configurations and locations of the studs, can obtain a lower torque index (or moment of force) in traction in boots with multi-studs designed for AG compared to other models with fewer studs. In addition, AG refers to a greater moment of force than NG, which will only be reduced when using footwear indicated for this terrain type. In NG soils, there is less rotational stiffness and, consequently, a lower overload index when compared to AG [11]. Boots appearing with six studs, generating considerable impact peaks in different traction tests carried out on AG, made a significant difference compared to designs with more studs, so their use is contraindicated on this type of terrain [12,13]. This differentiation of surfaces, linked mainly to the level of sports practice, indicates the need to analyze injuries in a sample that included both amateur and semi-professional soccer players.

This demonstrates the importance of choosing the right football boot, when considering reasons such as better performance at the expense of a higher rate of non-traumatic injuries [14]. For this reason, the main objective of this study was to analyze the relationship between sole pattern parameters of football boots with the frequency of injuries that occur in semiprofessional and amateur footballers. The authors hypothesize that abnormal distribution of the cleats on the sole of the shoe (unstable, insufficient in some areas or asymmetrical) and the absence of round cleats will result in a more significant number of non-traumatic injuries to the lower limb of the soccer player.

## 2. Materials and Methods

This study was conducted according to the guidelines of the Declaration of Helsinki and approved by the Ethics Committee of the University of Málaga (CEUMA Registration number: 56-2019-H). Researchers obtained informed consent from all subjects involved in the study. To conduct this cross-sectional study, 77 male football players voluntarily participated. The data collection occurred between January and July 2019 and the participants belonged to five teams (two semi-professional and three amateur teams). The team selection was established by mail contact and subsequently a meeting was arranged where the study objective was explained, followed by acceptance of their inclusion in the study. 

Participants met the following inclusion criteria: be over 18 years of age, play a minimum of 10 h per week, have no serious injuries or surgeries in the previous three months, belong to a federated football team for more than five years and give their informed consent.

### 2.1. Procedure

This study analysed data from each player’s medical history, including age, injuries, years of practice, foot posture index, field type and surface condition information. Visual Analogic Scale was used to analyze the degree of foot or ankle pain during sports and the associated location. A sports medicine specialist reviewed this condition (J.C.G.R.). The shoes a player wore during each football activity were analysed. Two podiatrists (A.G.P-A.C.D.) noted the shape and arrangement of the studs on the sole. The number of studs in each sole area was recorded, divided into external rearfoot, internal rearfoot, midfoot, fifth metatarsal, first metatarsal, central metatarsal and toes. Field condition was measured through field type (e.g., artificial vs. natural).

During the medical history data collection, an injury was defined as any acute reportable damage that occurred six months before data collection, required medical attention and restricted full sports participation in games or practices for one day or more. For this study, only lower extremity injuries, defined as any damage from the hips to the foot, were included in the research because these injuries are the most common in football. A data collection sheet was used to collect the history of injuries. Participants used an anatomical map on which they indicated the affected areas and described the type of injury, duration and date of the incident. This data was also cross-checked with the relevant medical team at each club. Each player’s total number of injuries was collected and categorized as trauma or non-contact injuries (including overuse injuries).

For descriptive purposes, the height (cm) and body mass (kg) were determined through a stadiometer and a precision scale (Seca, Hamburg, Germany) and the body mass index of the participants was calculated based on body mass and height (kg/m^2^). All measurements were taken with participants wearing underwear. Anthropometric measurements were taken following the guidelines of the International Society for the Advancement of Kinanthropometry [15].

### 2.2. Statistical Analysis

Statistical analysis was performed using IBM-SPSS Statistics v25.0 (IBM Corp. Released 2017; Armonk, NY, USA). Data are presented as means and standard deviations. Normality was analysed using the Kolmogorov-Smirnoff test. For the analysis of the relationship between the variables collected and the lesions, multiple regressions were performed with dichotomous variables regarding the presence or absence of injury, location and type of injury. 

A step-by-step multiple regression analysis was performed to determine the sole pattern variables (non-dependent) predictors of the number of injuries (dependent). The ROC curve procedure was used to find a cut-off point for the years of practice that could determine the presence of injury. In all these statistical tests, a significant value was considered when *p* < 0.05.

## 3. Results

The study included 46 semi-professional and 31 amateur-level football players. Summary of participants’ characteristics and variables related to sole pattern and injuries is shown in Table 1.

**Figure 1:**
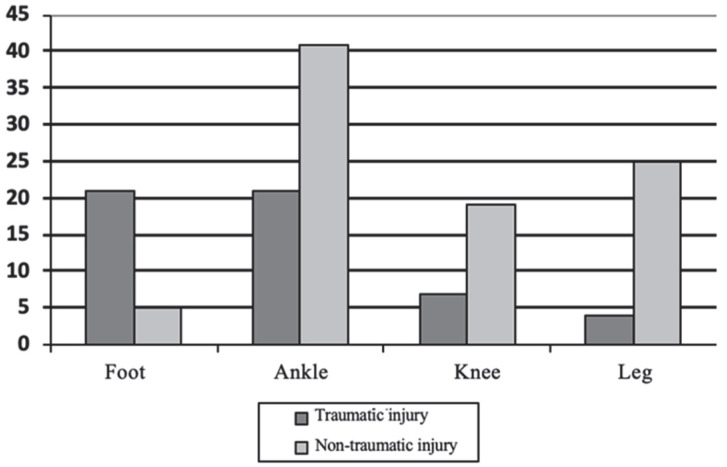
Frequency of lower limb injuries (*n* = 77).

The visual analogic score in semi-professional players was higher (2.05 ± 2.43) than in amateur players (1.00 ± 1.1). A total of 141 lesions were collected, equivalent to 1.81 injuries for each football player studied (*n* = 77). The total number of injuries suffered by football players was divided into four different areas of the lower limb and the origin, traumatic or non-traumatic (including overuse injuries). The ankle joint was affected in 43.97% of all registered injuries (*n* = 141), especially non-traumatic (29.07% of the total) (Figure 1).

Regarding the frequency of the types of studs, 57.8% of the studs were ovoid or circular (*n* = 44), the most used by most players. Ovoid studs are followed by triangular studs (*n* = 23) with a much lower 29.9% of the total. Boots with different types of studs or mixed and longitudinal were the least frequent, with 5.8% and 7.2%, respectively. The relationship between injury variables and the foot posture index, type of studs on the sole and surface did not show any significant results.

**Figure 2:**
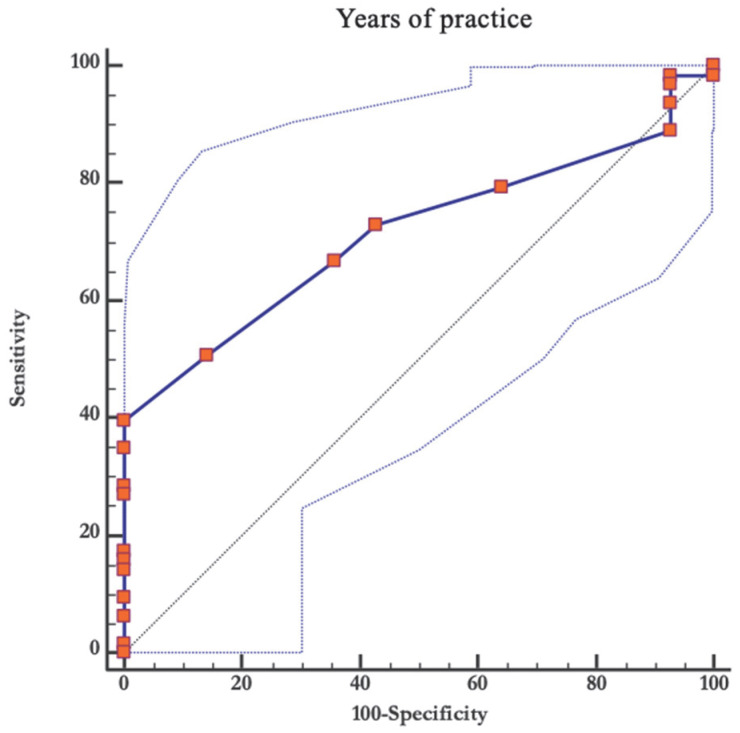
ROC curve analysis of the relationship between years of practice and appearance of injuries.

The result of the ROC curve indicated that the player’s years of practice could significantly (*p* < 0.05) predict the presence of lower limb injuries, with an area under the curve of 0.714, a regular-high accuracy. The curve coordinates indicate that an excellent cut-off value for maximizing sensitivity and specificity is 18 years or more of practice, where sensitivity = 39.68% and specificity = 100% (Figure 2).

Concerning the frequency of foot injuries, the multicollinearity variables were the number of medial studs in the rearfoot, lateral rear studs, toes and years of practice. The two-step of overload injuries prediction model (adjusted R^2^ = 0.388) initially included the number of medial rearfoot studs (B = 0.115, *p* = 0.000) and later the number of lateral rearfoot studs (B = −0.158, *p* = 0.000), explaining 38.8% of the variance for frequency of foot overload injuries. For the frequency of ankle overload injuries, the number of studs in the toes was a positive predictor of 4.4% of the frequency of injuries variance (B = 0.023, *p* = 0.046) and years of practice were a positive predictor of 5.3% of the frequency of lower limb injuries variance (R^2^ = 0.66, B = 0.085, *p* = 0.031) (Table 2).

## 4. Discussion

This study identified sole pattern parameters related to lower limb injuries, particularly to the foot and ankle regions. Specifically, a higher number of studs in the medial rearfoot and toes were predictors of foot and ankle overload injuries, respectively. The number of years of playing football was a predictor of lower limb injuries. No relationship between injuries and the using a different type of studs was found. 

The results obtained in this study placed the ankle as the most affected area in terms of injuries, mainly obtained without trauma, with a predominance of sprain as the most frequent injury. These data were supported by previous research on young amateur players and professional teams in FIFA and Olympic competitions [16]. In the case of the FIFA study, 17% of injuries of a total of 901 occurred in this area. A similar result was found in the study related to younger football players; 17% of the injuries were caused in the ankle out of 261 registered injuries. In studies carried out on women [17], ankle sprain was highlighted as the injury with the highest incidence, the majority being repetitive sprains and the majority of injuries due to trauma. In contrast, other studies on Premier League teams place the fifth metatarsal stress fracture as the most frequent injury to the foot and ankle [18].

Although the influence of shoe parameters on injuries in footballers seems clear [7,10,11,19], this study found no relationship between the type of cleat used and the injury history of footballers. Previous studies conclude that elongated studs can produce an abnormal increase in pressure under the lateral midfoot, which can cause injuries to the external metatarsals or be a substantial risk factor for injury [7,10]. The lack of relationship of recorded injuries to factors such as hours of practice, level of competition or playing surface might illustrate more clearly the fact that injuries recorded by soccer players stem from some characteristics of the soles of the boots and not from other causes.

Despite finding no association between stud type and injury, a relationship was established between the number of studs in various areas with the number of injuries sustained. Research analyzing the number of studs on the shoe’s sole focuses on the traction generated by the shoe on the ground and its relationship to lower limb injuries [19]. On the one hand, excessive rotational traction can lead to an anterior cruciate ligament injury in the knee [11]. On the other hand, footwear that does not provide enough traction force on the ground can cause slipping [6]. The resistance to these movements (turns or changes of direction) is considerably reduced using thirteen rounded studs [10,20] and the studied placement of the studs on the sole will improve the shoe’s traction during these specific movements [5]. A higher number of cleats on the medial rearfoot, combined with a lower number of cleats on the lateral rearfoot, was associated with the occurrence of overuse injuries in the player’s foot. These results may be due to the relationship of this stud’s distribution with a more supinated position of the rearfoot, which could overload lateral structures of the foot, such as the fourth and fifth metatarsals, peroneal tendons and lateral fascicle of the plantar fascia. Previous studies agree with these results, in which most injuries occurred in feet that were in a supinated position [18,21].

In the present study, we have verified that the most injured area of the lower limb in older football players is the ankle. The results indicate a relationship between the number of studs under the toes and ankle overuse injuries in football players. The results indicate that a higher number of studs under the toes may be associated with higher ankle overuse injuries in football players. This could be due to excessive traction during maximal plantar flexion, which compensatory movements in supination or pronation may accompany [21]. Therefore this implies that special attention is necessary for these players in preventing injuries of this type, either through the muscle strengthening described or preventive proprioception exercises [18].

Results for injuries occurring showed an increase in the probability of suffering them the greater the number of years of practice. This probability remains stable until 18 years of practice and from then on the probability of injury escalates to higher levels. To the authors’ knowledge, this relationship has not been previously studied. However, the influence of age on the likelihood of injury has been analyzed. Some studies agree on the increase in musculotendinous injuries from 23 [22], while others associate a higher number of fractures in players under 18 [23].

This study also presented some limitations. Selection bias based on recruitment strategy may have limitations in generalizing results and the cross-sectional design of the present study precluded causal inference. A longitudinal design or qualitative methodology must be used in future studies to investigate the possible causal relationship between sole patterns and lower limb injuries in football. This would have significant implications for consideration when football players plan choosing footwear for football practice. The distribution of shoe studs is an easy parameter to analyze that can be useful for any football trainer.

Authors should discuss the results and how they can be interpreted from the perspective of previous studies and of the working hypotheses. The findings and their implications should be discussed in the broadest context possible. Future research directions may also be highlighted.

## 5. Conclusions

This study described the predictive capacity of sole pattern characteristics concerning lower limb injuries in amateur and semi-professional footballers. Football boot variables associated with the number of studs (such as the number of studs in the rearfoot and toes) were associated with foot and ankle overload injuries. An increase in the number of medial rearfoot studs and a decrease in lateral rearfoot was associated with a higher appearance of foot injuries during running. The number of studs in the toes was a better predictor of ankle injuries. It is essential to consider the number of years of practice, as the probability of suffering injuries is higher in players playing football for over 18 years.

## Figures and Tables

**Table 1:** Descriptive analysis of the sample size (*n* = 77).

Variables	Total Sample Mean ± SD	Amateur Mean ± SD	Semi-Professional Mean ± SD
Age (years)	23.14 ± 4.8	24.87 ± 4.74	21.98 ± 4.52
Height (cm)	177.5 ± 0.07	175.68 ± 0.64	178.8 ± 0.6
Body mass (kg)	75.5 ± 10.64	77.36 ± 14.21	74.24 ± 7.24
Body mass index (kg/m^2^)	25.01 ± 3.19	25.05 ± 4.33	23.21 ± 1.8
Years of practice (years)	15.68 ± 4.65	15.97 ± 6.26	15.5 ± 3.2
Boot Replacement (months)	6.45 ± 5.07	10.1 ± 5.65	4 ± 2.6
Visual Analogic Scale	1.32 ± 1.72	1.77 ± 2.18	2.05 ± 2.43
Foot Posture Index (Right)	1.96 ± 2.91	2.58 ± 2.17	1.52 ± 3.27
Foot Posture Index (Left)	1.65 ± 3.07	2 ± 2.06	1.41 ± 3.6
Number of Boot Studs (per zone)			
Rearfoot	4.53 ± 1.09	4.54 ± 1.06	4.52 ± 1.11
Lateral rearfoot	2.23 ± 0.62	2.25 ± 0.6	2.22 ± 0.62
Medial rearfoot	2.17 ± 0.48	2.17 ± 0.38	2.17 ± 0.52
Midfoot	0.16 ± 0.5	0 ± 0	0.24 ± 0.6
5th metatarsal	2.26 ± 0.67	2.25 ± 0.73	2.26 ± 0.64
1st metatarsal	2.34 ± 1.01	2.04 ± 0.8	2.5 ± 1.07
Central metatarsals	1.59 ± 1.14	1.75 ± 1.03	1.5 ± 1.18
Toes	3.57 ± 1.75	4.21 ± 1.74	3.24 ± 1.67
Type of Surface			
Artificial Grass	38	20	18
Natural Grass	28	0	28
Land	11	11	0
Type of Stud			
Knife	6	2	4
Rounded	44	21	23
Triangular	23	8	15
Mixed	4	0	4

**Table 2:** Multiple linear regression model testing the significative association between injury variables with years of practice and foot shape characteristics.

	Years of Practice			Toes Studs			Medial Rearfoot Studs			Lateral Rearfoot Studs		
Dependent Variables	R^2^	B (SE)	*p*	R^2^	B (SE)	*p*	R^2^	B (SE)	*p*	R^2^	B (SE)	*p*
Total Injuries [lower limb]	0.66	0.085 (0.039)	0.031									
R^2^ adjusted		0.053										
Overload Injuries [ankle]				0.058	0.023 (0.011)	0.046						
R^2^ adjusted					0.044							
Overload Injuries [foot] Model 1							0.213	0.115 (0.027)	0.000			
R^2^ adjusted							0.201					
Overload Injuries [foot] Model 2							0.406	0.285 (0.043)	0.000	0.406	−0.158 (0.034)	0.000
R^2^ adjusted							0.388					

R^2^ = Coefficient of determination R square. B = Coefficient; SE = Standard error. Model 1: adjusted for medial rearfoot studs. Model 2: adjusted for medial rearfoot studs and lateral rearfoot studs.

## Data Availability

Not applicable.
